# Mevalonate Metabolism in Cancer Stemness and Trained Immunity

**DOI:** 10.3389/fonc.2018.00394

**Published:** 2018-09-20

**Authors:** Georg Gruenbacher, Martin Thurnher

**Affiliations:** Immunotherapy Research Unit, Department of Urology, Medical University of Innsbruck, Innsbruck, Austria

**Keywords:** epithelial mesenchymal transition (EMT), mevalonate metabolism, stemness, trained immunity, Wnt signaling

## Abstract

Mevalonate metabolism provides cancer and immune cells with diverse products to ensure cell functionality. Similar metabolic reprogramming that raises mevalonate metabolism to higher levels appears to drive both, epithelial mesenchymal transition (EMT) of cancer cells, a reverse differentiation program that generates cancer cells with stem cell properties, and immune cell training for increased responsiveness to secondary stimulation. In this review, we address how mevalonate metabolism supports cancer development and stemness on the one hand, and on the other promotes immune responsiveness. In view of this dual nature of mevalonate metabolism, strategies to manipulate this metabolic pathway as part of anti-cancer therapies require careful analysis of risks versus benefits.

## Introduction

**Figure 1:**
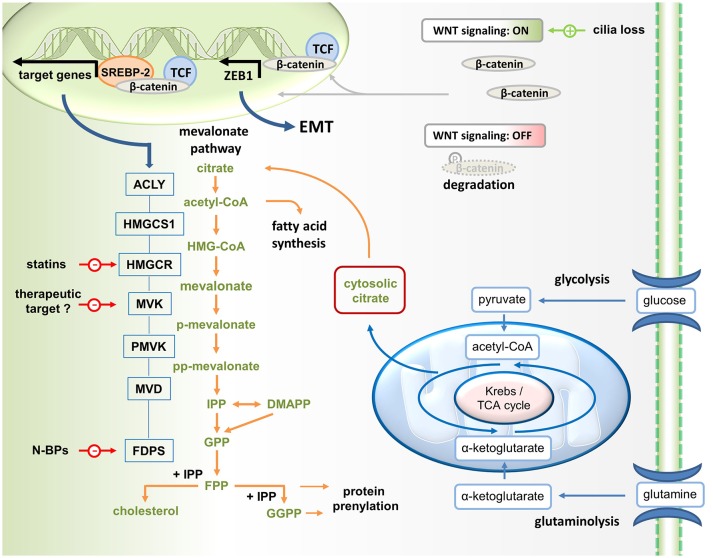
Wnt/ß-catenin driven mevalonate metabolism promotes EMT. Mevalonate metabolism is fueled by glycolysis. Glucose-derived pyruvate enters the TCA cycle. Since some citrate is exported into the cytosol for lipogenesis (i.e., mevalonate metabolism and fatty acid synthesis), glutaminolysis generates α-ketoglutarate to replenish TCA cycle intermediates. Canonical Wnt signaling, which can be activated for instance in response to cilia loss, promotes the translocation of β-catenin to the nucleus, where it acts as coactivator of TCF factors in the transcription of target genes. In addition, ß-catenin may stimulate mevalonate metabolism by forming a complex with SREBP2 on the promoters of mevalonate pathway genes, including HMGCR (HMG-CoA reductase, the target of statins). In addition, β-catenin/TCF4 binds directly to the promoter of ZEB1, a key inducer of EMT, and activates its transcription. N-BPs, nitrogen-containing bisphosphonates.

The metabolic reprogramming that drives both, the malignant process and immunity, results in increased glycolysis and glutaminolysis, which fuel the mitochondrial citrate cycle to generate ATP (Figure 1). Such a metabolic constellation also facilitates the partial export of mitochondrial citrate into the cytosol. After hydrolysis by ATP citrate lyase (ACLY), acetyl-CoA becomes available for mevalonate generation and metabolism as well as for fatty acid synthesis (lipogenesis), a process that is driven by signaling through AKT and mTOR (mechanistic target of rapamycin). While AKT and mTOR generally promote glycolysis-driven lipogenesis (1), AKT has been reported to specifically control ACLY (2, 3). These lipogenic pathways provide cholesterol and fatty acids for membrane biogenesis and are therefore essential for cancer cell growth and proliferation (4–6). The expression of mevalonate pathway genes is governed by the sterol regulatory element-binding protein (SREBP) transcription factors. SREBPs have been shown to stimulate gene expression in the lipogenic pathways (7), including ACLY and HMGCR (HMG-CoA reductase) (Figure 1).

## Mevalonate metabolism promotes EMT and tumorigenesis

Metabolic reprogramming toward increased mevalonate pathway activity may also be associated with EMT (8–11). EMT, which describes the ability of epithelial cells to transdifferentiate into mesenchymal cells, generates cells with stem cell features and migratory capacity (9). In malignant cells, EMT produces cancer stem cells with an invasive phenotype and thus promotes metastasis formation (12).

A phenotypic hallmark of EMT is the loss of E-cadherin expression. EMT-inducing transcription factors such as Snail1, Snail2 (Slug), ZEB1, ZEB2, and TCF3 as well as KLF8 (Kruppel-like factor 8) can directly suppress E-cadherin expression. Growth factor signaling (TGFß, FGF, EGF, HGF) as well as Wnt/ß-catenin signaling, Notch signaling and hypoxia can induce EMT. CD44 expression has also been correlated with EMT, thereby promoting tumor invasion, metastasis, chemoresistance and recurrence (13, 14). As a cell surface protein CD44 interacts with various receptor tyrosine kinases to modulate cellular signaling (15). Oncogenic Ras signaling has been shown to promote CD44 splicing (16) leading to the expression of distinct CD44 isoforms in proliferating cells and tumors. Importantly, a switch in alternative splicing of CD44 has been reported to be essential for EMT and especially CD44v isoforms have been identified as cancer stem cell surface markers (17).

A study of the epithelial membrane lipidome revealed that epithelial cells undergoing EMT increase cholesterol levels (18), indicative of enhanced mevalonate pathway activity. As a consequence, the resulting mesenchymal cells were more sensitive to statin treatment, which also reduced membrane fluidity and migratory capacity as well as metastatic potential (19), confirming the importance of mevalonate metabolism in EMT.

In addition to cholesterol, mevalonate metabolism provides farnesyl diphosphate (FPP) and geranylgeranyl diphosphate (GGPP), which are the activated forms of farnesyl and geranylgeranyl serving as substrates of the respective transferases in post-translational protein prenylation (Figure 1). Farnesylation or geranylgeranylation is required for the biological activity of many Ras superfamily members.

The role of the RAS superfamily of GTPases in tumorigenesis is well established (20). Uncontrolled flux through the mevalonate pathway promotes sustained Ras protein activation via constitutive prenylation. The tumor suppressor p53 controls mevalonate metabolism. However, gain-of-function p53 mutations may cause p53-mediated enhancement of mevalonate metabolism and subsequently also of protein prenylation, facilitating malignant transformation (21, 22). Sustained accumulation of mutant p53 mediated by geranylgeranylated RhoA (23) generates a self-perpetuating dynamic that may further promote malignancy.

A recent study by Deng et al. reported that targeted disruption of cilium biogenesis (ciliogenesis) sensitizes normal cells (pancreatic cells (HPDE6C7) and NIH3T3 cells) to oncogene (K-Ras)-driven malignant transformation through activating the mevalonate pathway (24). The authors observed disrupted ciliogenesis in mouse (breast and liver) as well as in human (lung, ovary, and pancreas) cancer cell lines (24). Moreover, cilia loss was detected in a mouse model of a precursor of pancreatic ductal adenocarcinoma. These observations were consistent with previous reports of ciliogenesis defects in human glioma, breast, and kidney cancer (25, 26).

Deng et al. found that cilia loss significantly increased the expression of ACLY, which determines the availability of cytosolic acetyl-CoA, the precursor to mevalonate (Figure 1). In addition, cilia loss promotes expression of several mevalonate pathway genes including HMGCR encoding MHG-CoA reductase, which catalyzes mevalonate formation. Statin-mediated MHG-CoA reductase inhibition could effectively reverse the oncogenic phenotype induced by cilia disruption. Cells with disrupted ciliogenesis appeared to be more dependent on mevalonate metabolism for survival and, thus, were more sensitive to statin treatment. This is in line with another recent study reporting that sensitization to statin treatment is related to H-Ras-induced EMT through activation of ZEB1 (27), which is a key inducer of epithelial transdifferentiation.

In the study by Deng et al., the reprogramming in response to cilia loss included activation of the Wnt–β-catenin signaling pathway (24), which also induces EMT. In the canonical Wnt pathway, stimulation promotes the translocation of β-catenin to the nucleus, where it acts as coactivator of T-cell and lymphoid enhancer (TCF–LEF) factors in the transcriptional activation of target genes (28). The potentially oncogenic Wnt–β-catenin signaling pathway triggers an EMT via ZEB1 (29). Deng et al. reported that several genes encoding mevalonate pathway enzymes, including HMGCR which encodes the pathway-initiating enzyme HMG-CoA reductase, turned out to be direct target genes of β-catenin–TCF signaling (Figure 1). Mechanistically, Wnt–β-catenin signaling appeared to stimulate mevalonate metabolism by complex formation between β-catenin and SREBP2 on the promoters of the genes involved in the mevalonate pathway.

Taken together, these observations suggested that oncogenic signaling, for instance via K-Ras, can suppress ciliogenesis resulting in a cellular reprogramming that is characterized by β-catenin–TCF driven mevalonate metabolism and EMT-like differentiation. In accordance with such a view, deficiency of primary cilia in kidney epithelial cells has recently been shown to induce EMT (30).

## Mevalonate metabolism promotes cancer stemness

The protooncogene MYC is well known to induce metabolic reprogramming, including stimulation of lipogenesis (31). Myc-driven mevalonate metabolism has recently been shown to maintain brain tumor-initiating cells (BTICs) (32). Such stem-like cancer cells share signaling and metabolic preferences with cancer cells upon EMT (10). In BTIC models numerous mevalonate pathway genes were activated. Consistent with the importance of mevalonate metabolism for stemness, mevalonate pathway genes were suppressed when differentiation was induced in BTICs. Likewise, inactivation of HMG-CoA reductase, the mevalonate-generating enzyme that initiates the pathway, either by attenuation of gene expression or by statin-mediated enzyme inhibition reduced self-renewal capacity and tumorigenicity of these cells. In addition, statin treatment also reduced Myc expression in BTICs (32).

Myc is a main driver of stemness in different types of cancer (33). Myc promotes the formation of tumor spheres (34), which are considered a surrogate marker of self-renewal capacity (35). Sphere formation initially requires detachment from a tissue and its extracellular matrix (ECM), which is mediated by the downregulation of genes encoding distinct integrins involved in cell-to-cell contact as well as by the upregulation of genes encoding matrix metallopetidases, thus promoting ECM degradation. This process of spinning off and establishing an independent spherical colony through anchorage-independent growth is reminiscent of the EMT-driven initiation of metastasis. Importantly, Myc-driven sphere formation has been shown to be associated with increased expression of sterol regulatory element binding factors and transcriptional activation of cholesterol synthesis in the mevalonate pathway (34). Collectively, these findings indicate that mevalonate metabolism drives EMT-induced stemness.

## Mevalonate metabolism enhances innate immunity

A metabolic reprogramming toward enhanced flux through the mevalonate pathway similar to that observed in cancer cells and cancer stem cells has been observed in innate immune cells, which as a consequence exhibit increased responsiveness. As a metabolic pathway that supports cell growth and proliferation, mevalonate metabolism is already required during macrophage development. M-CSF, a main driver of myelopoiesis, promotes glycolysis, which in turn stimulates Myc-driven activation of lipogenic pathways (36). Such M-CSF induced metabolic reprogramming included the transcription of multiple genes involved in mevalonate generation and in cholesterol biosynthesis. Conversely, attenuation of mevalonate metabolism impaired myelopoiesis (36).

In addition to its role in macrophage development, mevalonate metabolism is crucial for the effector function of M1 macrophages, which exhibit strong tumoricidal activity in response to classical activation by the Th1 cytokine interferon-γ (IFN-γ) plus lipopolysaccharide (LPS) (37). In M1 macrophages, GM-CSF increases glycolytic capacity. GM-CSF thus primes M1 macrophages for a particularly strong glycolytic response upon LPS stimulation, also resulting in significantly higher levels of pro-inflammatory cytokines. Since glycolysis fuels mevalonate metabolism, it was not surprising that GM-CSF primed macrophages also contained higher levels of acetyl-CoA, the precursor to mevalonate, and displayed increased expression of HMG-CoA reductase, the mevalonate-generating enzyme, which is inhibited by statins. The ability of statins to inhibit cytokine responses of M1 macrophages and to prevent the priming effect of GM-CSF highlights the role of mevalonate metabolism in M1 macrophage function.

Both, M-CSF and GM-CSF have also been implicated in the induction of EMT. For instance, M-CSF can promote EMT of lung cancer cells via canonical Wnt signaling (38). An EMT-inducing potential of GM-CSF has been reported in colon cancer. GM-CSF-trained cancer cells displayed mesenchymal features, including motility and chemoresistance, and ZEB1 as well as MAPK/ERK signaling were identified as critical factors of GM-CSF-driven EMT (39).

Although memory has long been considered a specific feature of adaptive immune cells, i.e. B and T cells, recent work has demonstrated that innate immune cells can also develop a form of memory after microbial stimulation during infections or vaccinations (40). Similar to antigen priming of lymphocytes, the repetitive stimulation of monocytes with Bacillus Calmette–Guérin (BCG) or β-glucan via toll-like receptors facilitated the increased production of inflammatory cytokines and reactive oxygen intermediates in response to subsequent challenges. Akt–mTOR-driven metabolic reprogramming toward enhanced flux through the mevalonate pathway was identified as the underlying mechanism that drives improved innate immunity.

Although initially developed as a tuberculosis vaccine, BCG treatment is also among the most effective cancer immunotherapies, and in high-risk, non-muscle-invasive bladder cancer, the repetitive intravesical instillation of BCG is still the standard adjuvant treatment according to the European Association of Urology (EAU) guidelines (41, 42). The relevance of mevalonate metabolism in BCG-induced immune training was demonstrated when statins were shown to impair both, the memory of monocytes in the *in vitro* model (40) and the clinical outcome of BCG treatment for bladder cancer (43).

A recent study performed in mice but with important clinical implications demonstrated that access of BCG to the bone marrow enhances myelopoiesis and educates hematopoietic stem cells to generate trained monocytes, which are highly protective against *M. tuberculosis* infection (44). Likewise, administration of ß-glucan to mice was shown to also enhance myelopoiesis in the bone marrow via local production of IL-1ß and GM-CSF (45). In expanding progenitors, several mevalonate pathway genes were significantly upregulated, including HMGCR, encoding HMG-CoA reductase. Intriguingly, ß-glucan-enhanced myelopoiesis also turned out to be protective to chemotherapy-induced myeloablation.

Statins are HMG-CoA reductase inhibitors and thus block the production of mevalonate suggesting that lack of the precursor prevents the generation of immunity-promoting products such as cholesterol or the protein prenylation substrates FPP and GGPP. However, mevalonate itself was recently identified as a key mediator of innate immune training. Mevalonate was found to improve the functionality of the insulin-like growth factor 1 (IGF1) receptor, leading to the activation of mTOR and subsequent histone modifications in inflammatory pathways (46). Importantly, IGF1 receptor signaling also induces EMT and contributes to metastasis and drug resistance in different types of tumors (47), indicating parallels between EMT and trained innate immunity.

Once mevalonate is formed by HMG-CoA reductase, the pathway continues by mevalonate phosphorylation through mevalonate kinase (MVK). In humans, MVK mutation may strongly attenuate enzyme activity, leading to hyper IgD syndrome (HIDS). This block in mevalonate metabolism is well known to result in sterile inflammation including periodic fever attacks. A novel aspect, however, is that accumulation of mevalonate induces a trained immunity phenotype in monocytes from these patients via improved IGF1 receptor functionality similar to BCG or ß-glucan-induced immune training (46). Transcriptional profiling of trained monocytes from HIDS patients revealed upregulation of the EMT-inducing transcription factor ZEB1 and of the EMT-associated cancer stem cell promoting chemokine IL8 (46, 48, 49).

Another phenotypic marker of EMT is the increased expression of the neural cell adhesion molecule (NCAM) (8, 50). NCAM is expressed by neurons and glia as well as skeletal muscle cells. The same protein, however, is also expressed in the hematopoietic system, where it is referred to as CD56. Among blood cells, CD56 is mainly associated with NK cells (51), subsets of γδ T cells (52) and CD8+ T cells (53) as well as on a subset of dendritic cells (52, 54, 55). Intriguingly, in all cases increased CD56 (NCAM) expression was associated with enhanced cellular functionality.

Collectively, these observations suggest that an EMT-like reprogramming toward enhanced flux through the mevalonate pathway similar to that observed in cancer cells may also promote innate immunity.

## Enhanced mevalonate metabolism in cancer cells alerts γδ T cells

As a growth and proliferation-promoting pathway mevalonate metabolism also contributes to tumorigenesis (21, 22) and immune surveillance of this potentially oncogenic pathway is therefore desirable. A subset of human γδ T cells, which are innate-like T cells with strong antitumor activity, is capable of recognizing and killing cancer cells with a hyperactive mevalonate pathway (56). Vγ9Vδ2 T cells, which represent the main γδ T cell population in human peripheral blood, recognize small isoprenoid phosphoantigens (57, 58). The mevalonate pathway intermediate isopentenyl diphosphate (IPP) is a strong agonist of γδ T cells expressing the Vγ9Vδ2 T cell receptor and IPP-activated Vγ9Vδ2 T cells can proliferate, produce effector cytokines such as interferon (IFN)-γ and display natural killer cell-like cytotoxicity, especially in the presence of interleukin-2 (IL-2). Nitrogen-containing bisphosphonates (N-BPs) are another class of mevalonate pathway manipulating drugs, which inhibit FPP synthase (Figure 1). Although N-BPs have been developed for the treatment of osteoporosis, N-BPs also have a long history in cancer treatment (59). N-BPs may exhibit direct antitumor effects but may also generate therapeutic effects through immune activation. Treatment of cancer cells or antigen-presenting cells with N-BPs causes IPP accumulation in these cells (60). As a consequence these cells acquire the capacity to activate Vγ9Vδ2 T cells. In addition to IPP accumulation, N-BP-mediated FPP synthase inhibition also causes downstream depletion of FPP and GGPP, resulting in an inflammatory response that depends on caspase-1 activated IL-1ß and IL-18 (54, 55). These cytokines can costimulate the IPP-driven activation of Vγ9Vδ2 T cells (52) and may facilitate concomitant NK cell activation (55). Similar to N-BP treatment, ectopic expression of HMGCR (Figure 1) in cancer cells increases the flux through the mevalonate pathway and also renders these cells targets of Vγ9Vδ2 T cells (60). Collectively, these and other findings indicate that Vγ9Vδ2 T cells participate in immune surveillance of the potentially oncogenic mevalonate pathway.

## Mevalonate metabolism may also enhance adaptive immunity

The metabolic reprogramming toward increased glycolysis during T cell activation is controlled by Myc (61) and ensures the availability of building blocks required for cell growth, proliferation and effector function. Myc-driven glycolysis fuels lipogenesis. At the transcriptional level, T cell receptor triggering results in the SREBP2-dependent activation of genes encoding enzymes involved in mevalonate generation and metabolism (7). In contrast to activated T cells, which preferentially engage glycolysis, memory T cells appear to rely on fatty acid oxidation (FAO) (62). As resting cells, memory T cells use FAO-derived ATP for survival. The role of the mevalonate pathway in this particular metabolic constellation is unclear. Fatty acid breakdown generates acetyl-CoA, which can be oxidized in the mitochondria or serve as a substrate in protein acetylation and, in principle, also as a precursor in mevalonate synthesis. Further work is needed to clarify the role of mevalonate metabolism in memory T cells.

The transcription factor GATA-3 has been shown to control Myc expression and to be critical for peripheral T cell maintenance through IL-7 signaling (63). The peripheral maintenance of GATA-3 deficient CD8 T cells was impaired with reduced IL-7 receptor (IL-7R) expression. These findings indicated that GATA-3 is required for IL-7R expression in CD62L^hi^CD44^hi^ central memory CD8 T cells. Both, CD44 and IL-7R (CD127) have been described as markers of EMT (13, 64). Moreover, CD44 expression has been reported to function as a sensitive reporter of tonic mTOR-S6 kinase signaling (65), a signaling pathway well known to promote mevalonate metabolism (66). GATA-3 robustly upregulates Myc (63), which drives the metabolic reprogramming associated with EMT. Canonical Wnt signaling, which promotes EMT and mevalonate metabolism, also enhances GATA-3 expression (67), altogether suggesting that mevalonate metabolism may also play a role in memory T cells.

## Concluding remarks

It is meanwhile well accepted that glycolysis-driven mevalonate metabolism governs the functions of activated cells, including cancer and immune cells. To target one population without impairing the other represents a great therapeutic challenge. Among mevalonate pathway inhibitors (20), statins inhibit the pathway early on and actually prevent mevalonate generation. The increase of mevalonate pathway activity that occurs during EMT sensitizes cancer cells to the antitumor effects of statins (19). While statins may thus have direct antitumor effects, also by reducing cancer stemness (32), they will concomitantly also suppress innate immunity and, in particular, prevent the phenomenon of trained immunity in monocytes that critically depends on mevalonate (46). In addition, statins preclude accumulation of IPP and, as a consequence, they prevent Vγ9Vδ2 T cells from exercising their cancer surveillance function (60). The recent observation that HIDS patients, which lack MVK, have a trained immunity phenotype (46), suggests that MVK might be an attractive target. Pharmacological inhibition of MVK causes mevalonate accumulation and thus immune training of monocytes. At the same time, MVK inhibition would deplete downstream metabolites such as FPP and GGPP in cancer cells. This effect will attenuate protein prenylation of multiple Ras family members and thus antagonize cancer stemness. However, just like the statins, MVK inhibition would prevent of IPP accumulation and γδ T cell activation. Still left to be evaluated are the N-BPs, which inhibit protein prenylation (20), generate immune training effects (54) and expand Vγ9Vδ2 T cells (68). Appropriate administration and combination, for instance with cytokines, may therefore lead to regimen that exploit the full potential of N-BPs.

## Author contributions

MT gathered information and wrote the first draft of the manuscript. GG participated in writing and editing of the final manuscript and prepared the figure. Both authors read and approved the submitted version.

### Conflict of interest statement

The authors declare that the research was conducted in the absence of any commercial or financial relationships that could be construed as a potential conflict of interest. The handling editor declared a shared affiliation, though no other collaboration, with the authors MT and GG.
